# Use of plasma rich in growth factors (PRGF-Endoret®) fibrin membrane to cover corneal dellen

**DOI:** 10.22336/rjo.2021.60

**Published:** 2021

**Authors:** Miriam Rahhal-Ortuño, Alex Samir Fernández-Santodomingo, Clara Martínez-Rubio, Honorio Barranco-González, Inmaculada Almor-Palacios, Ana Rodrigo-Hernández

**Affiliations:** * Department of Ophthalmology, Hospital Universitari i Politecnic La Fe, Valencia, Spain

**Keywords:** dellen, papilloma, Plasma rich in growth factors

## Abstract

Corneal dellen appeared as a complication after perilimbal conjunctival papilloma dissection in a six-year-old patient. Our purpose was to describe the use of plasma rich in growth factors (PRGF) fibrin membrane in covering the corneal defect after conventional medical treatment failure. PRGF fibrin membrane is an interesting therapeutical option to consider not only in adult patients, but also in children.

## Introduction

Conjunctival papilloma are benign squamous cell tumors that are related to human papilloma virus infection. They can be asymptomatic or give rise to ocular surface disturbances causing dryness and conjunctival hemorrhages among others. Papilloma are characterized by small epithelium projections with underlying vascular loops. However, their clinical appearance is sometimes not sufficient for diagnosis, the histological examination being the gold standard for this purpose [**1**]. 

Corneal dellen is a possible complication that may appear after ocular surgeries involving perilimbal conjunctiva such as pterygium excision [**2**], strabismus surgery [**3**], or papilloma excision. Ultimately, it can give rise to corneal perforation [**2**], being therefore its treatment of great importance.

## Case presentation

A six-year-old Caucasian male patient was referred to our Ophthalmology Department due to the presence of a perilimbal tumoral lesion in his left eye, which was causing eye discomfort. The lesion affected the temporal conjunctiva and invaded cornea without reaching visual axis. 

Visual acuity was 1.0 in both eyes. Anterior segment slit lamp examination showed no pathological signs apart from the conjunctival lesion described and intraocular pressure was not altered. Fundus examination revealed no abnormalities. 

Surgical excision was decided in order to histologically examine the lesion.

The surgical procedure was carried out under general anaesthesia using a crescent blade knife to perform lamellar dissection. Histological examination revealed the lesion corresponded to a conjunctival papilloma. 

Visual acuity and intraocular pressure did not vary after the surgical procedure. 

**Fig. 1:**
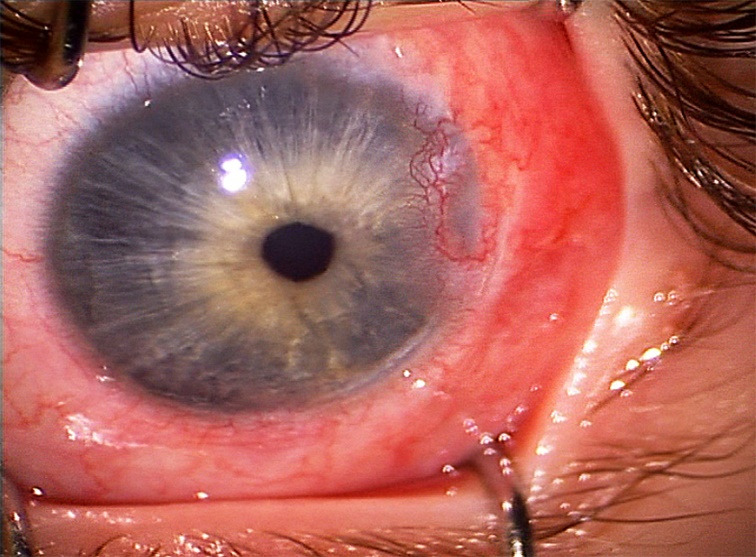
Intraoperative image showing temporal corneal dellen associated to neovascularization

During the fourth week follow-up, the patient showed increased hyperaemia and referred severe eye discomfort. A corneal dellen was appreciated associated to neovascularization affecting the temporal peripheral cornea of the patient’s left eye (**Fig. 1**). Lubricant eye drops and gels associated to prolonged occlusions were prescribed for over six weeks with no satisfactory results. As corneal dellen did not resolve with classical therapeutic options, a surgical approach was decided.

Under general anaesthesia, temporal peritomy was performed and the conjunctiva was dissected in this area. The PRGF fibrin membrane previously prepared, was placed under the conjunctiva and over the corneal defect, then trimmed down to adapt it to the area-to-cover and sutured.

In order to prepare the PRGF membrane, plasma obtained after centrifuging the patient’s blood was combined with calcium chloride and thrombin. Incubation at high temperature allowed soluble plasma fibrinogen to be converted into an insoluble fibrin membrane. 

The patients’ blood sample was also used to manufacture PRGF eyedrops, which were prescribed four times a day as part of the postoperative treatment for two months. In order to prepare them, the blood sample was placed into tubes with sodium citrate, which were then centrifuged at room temperature. The plasma column that was formed over the buffy coat after centrifugation was collected. The resulting product was filtered and frozen until use. 

**Fig. 2:**
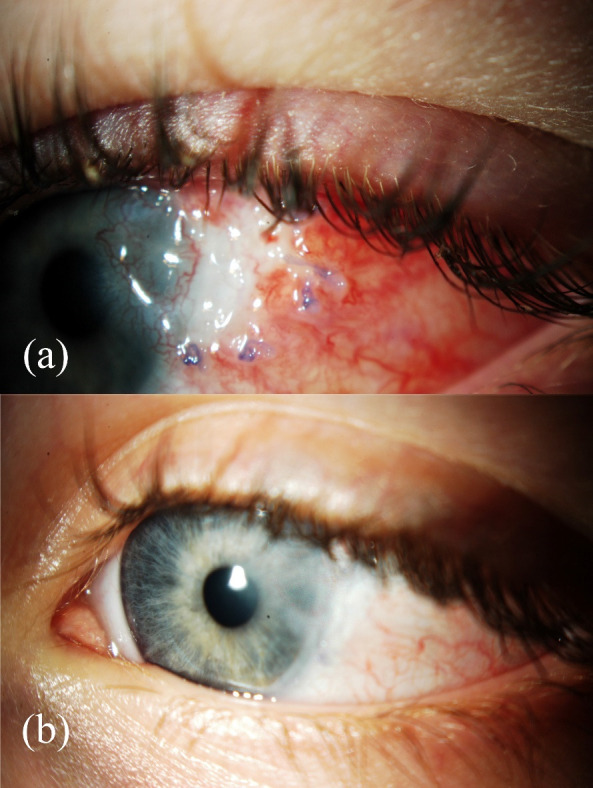
Postoperative follow-up. **(a)** One week after surgery. **(b)** One month after surgery showing resolution of previous defect

Weekly follow-ups showed reabsorption of the fibrin membrane after three weeks and ophthalmological examination revealed corneal dellen resolution one month after the surgical procedure was undergone (**Fig. 2**).

No tumoral regression has been observed to date.

## Discussion

Plasma rich in growth factors (PRGF) is a particular type of platelet rich plasma manufactured with the Endoret® (BTI Biotechnology Institute, S.L., Miñano, Álava, Spain) system [**4**]. It is a blood-based therapeutic technology that aids tissue repair and regeneration. It is obtained from the patient’s own blood that undergoes a centrifugation process and posterior activation of the plasma fraction with calcium chloride so that growth factors including epidermal growth factor (EGF), platelet-derived growth factor AB (PDGF-AB), transforming growth factor-beta 1 (TGF-ß1), vascular endothelial growth factor (VEGF), insulin-like growth factor type I (IGF-I) and fibroblast growth factor (FGF) are released [**5**]. It can then be used as eye drops or as a fibrin membrane that can be sutured over the defect to treat [**6**]. 

In vitro and in vivo studies have demonstrated its efficacy and shown better results than when autologous serum is used in terms of corneal wound closure, corneal epithelial cell proliferation and migration [**7**].

Its bacteriostatic activity and anti-fibrotic and anti-inflammatory properties also aid in tissue regeneration. Corneal scar formation is significantly decreased as PRGF avoids stromal fibroblasts to differentiate into myofibroblasts, which persist after wound healing, leading to haze and fibrotic scars [**7**]. 

It has been shown that fibrin present in PRGF membranes bridges tissue gaps and mediates in cell proliferation and migration, being successfully used to treat deep corneal ulcers and as a bio-adhesive material to attach corneal flaps in lamellar keratoplasty in preclinical studies [**6**]. 

Use of autologous platelet-rich plasma was first described by Alio et al. in corneal ulcers, achieving satisfactory results in terms of pain, inflammation, and wound healing [**8**]. PRGF ophthalmological uses described after this, include dry eye syndrome, persistent epithelial defect, and neurotrophic keratopathy [**9**]. Most reports in ophthalmological literature describe PRGF as eye drops, being its use as a fibrin membrane less reported. Keratoplasty, limbal stem cell transplantation and amniotic membrane are still the most often described therapeutic options for ocular surface reconstruction [**7**]. 

## Conclusion

To our knowledge, this is the first reported case of PRGF membrane used in children and as an adjuvant treatment after limbal tumoral lesions’ excision.

**Conflict of Interest statement**

Authors state no conflict of interest.

**Informed Consent and Human and Animal Rights statement**

Consent was gathered from the patient’s tutors in order to obtain and publish these images.

**Authorization for the use of human subjects**

Ethical approval: The research related to human use complies with all the relevant national regulations, institutional policies, is in accordance with the tenets of the Helsinki Declaration, and has been approved by the review board of Hospital Universitari i Politecnic La Fe, Valencia, Spain.

**Acknowledgements**

None.

**Sources of Funding**

There are no funders to report for this submission.

**Disclosures**

None.
